# DNA Methylation and Subgenome Dominance Reveal the Role of Lipid Metabolism in Jinhu Grouper Heterosis

**DOI:** 10.3390/ijms25179740

**Published:** 2024-09-09

**Authors:** Yang Liu, Linna Wang, Zhentong Li, Linlin Li, Shuai Chen, Pengfei Duan, Xinyi Wang, Yishu Qiu, Xiaoyu Ding, Jinzhi Su, Yuan Deng, Yongsheng Tian

**Affiliations:** 1 State Key Laboratory of Mariculture Biobreeding and Sustainable Goods, Yellow Sea Fisheries Research Institute, Chinese Academy of Fishery Sciences, Qingdao 266071, China; yangliu@ysfri.ac.cn (Y.L.); wangln@ysfri.ac.cn (L.W.); lizt@ysfri.ac.cn (Z.L.); lill@ysfri.ac.cn (L.L.); fisherman923@163.com (S.C.); duanpf_gs@163.com (P.D.); wxy229746642@163.com (X.W.); yishuqiu97@163.com (Y.Q.); dingxy0503@163.com (X.D.); s17852137035@163.com (J.S.); dyadela@163.com (Y.D.); 2 Laboratory for Marine Fisheries Science and Food Production Processes, Qingdao Marine Science and Technology Center, Qingdao 266237, China; 3 Hainan Innovation Research Institute, Chinese Academy of Fishery Sciences, Sanya 572000, China

**Keywords:** Jinhu grouper (*Epinephelus fuscoguttatus* ♀ × *E. tukula* ♂), methylome, transcriptome, allele-specific expression (ASE), growth heterosis, lipid metabolism

## Abstract

Heterosis of growth traits in economic fish has benefited the production of aquaculture for many years, yet its genetic and molecular basis has remained obscure. Nowadays, a new germplasm of hybrid Jinhu grouper (*Epinephelus fuscoguttatus* ♀ × *E. tukula* ♂), abbreviated as EFT, exhibiting paternal-biased growth heterosis, has provided an excellent model for investigating the potential regulatory mechanisms of heterosis. We integrated transcriptome and methylome to unravel the changes of gene expression, epigenetic modification, and subgenome dominance in EFT compared with maternal *E. fuscoguttatus*. Integration analyses showed that the heterotic hybrids showed lower genomic DNA methylation levels than the purebred parent, and the up-regulated genes were mostly DNA hypomethylation. Furthermore, allele-specific expression (ASE) detected paternal subgenome dominance-regulated paternal-biased heterosis, and paternal bias differentially expressed genes (DEGs) were wholly up-regulated in the muscle. Multi-omics results highlighted the role of lipid metabolism, particularly “Fatty acid synthesis”, “EPA biosynthesis”, and “Signaling lipids”, in EFT heterosis formation. Coherently, our studies have proved that the eicosapentaenoic acid (EPA) of EFT was greater than that of maternal *E. fuscoguttatus* (8.46% vs. 7.46%). Finally, we constructed a potential regulatory network for control of the heterosis formation in EFT. Among them, *fasn*, *pparg*, *dgat1*, *igf1*, *pomca*, *fgf8a*, and *fgfr4* were identified as key genes. Our results provide new and valuable clues for understanding paternal-biased growth heterosis in EFT, taking a significant step towards the molecular basis of heterosis.

## 1. Introduction

Hybridization is a driving force of genomic evolution and speciation resulting from the merging of two divergent genomes, which can lead to immediate and profound genome modifications, such as structural variation, epigenetic diversity, gene expression bias, and transposon activities [1,2]. Hybrid genomes may produce novel genetic and phenotypic variations that can contribute to heterosis, such as fast growth, disease resistance, and stress tolerance [3]. However, heterosis is uncertain, depending on the parents. For example, the channel catfish (*Ictalurus punctatus*, female) × blue catfish (*I. furcatus*, male) hybrid shows superior growth characteristics than its parents, but no such growth vigor could be found in mating the male channel catfish with the female blue catfish [4]. In hybrid tilapia, Nile tilapia (*Oreochromis niloticus*, female) × blue tilapia (*O. aureus*, male) hybrid shows growth heterosis, but blue tilapia (female) × Nile tilapia (male) hybrid failed to show this [5]. Although these hybrid lineages of the reciprocal crosses harbor the same genomic origins, they show opposite phenotypic variation. Thus, exploring the molecular basis of heterosis in hybrids is crucial for hybridization speciation and cross breeding.

Groupers of the family Epinephelidae, as protogynous hermaphroditic teleosts, are widely distributed in tropical and subtropical oceans. They comprise over 160 species, providing abundant genetic resources for cross breeding [6]. So far, three new hybrid groupers have received approval from the Chinese government: Hulong grouper (*Epinephelus fuscoguttatus* ♀ × *E. lanceolatus* ♂) [7], Yunlong grouper (*E. moara* ♀ × *E. lanceolatus* ♂) [8], and Jinhu grouper (*E. fuscoguttatus* ♀ × *E. tukula* ♂), abbreviated as EFT below [9]. These hybrids exhibit prominent heterosis in growth rate, feed conversion efficiency, and survival rate that enhances the yield of traditional grouper farming. Based on the China Fishery Statistical Yearbook, grouper production has reached 205,816 tons in 2022, and hybrid varieties constitute more than 70% of the total harvest [10]. We found that the formation of superior growth heterosis generally requires a large grouper as a male parent. For example, the giant grouper (*E. lanceolatus*) body mass can reach up to 3 kg in the first year, and with a maximum body weight above 400 kg and length over 200 cm [11,12]. The potato grouper (*E. tukula*) can reach at least 150 cm in total length and 90 kg in weight [13]. But giant grouper and potato grouper are difficult to artificially reproduce; thus, their sperm has been widely used in cross breeding to transfer the genome of rapid growth to hybrid offspring. The newly formed hybrid EFT exhibits the heterosis of fast growth, low-temperature resistance (9 °C), and low-oxygen tolerance (0.24 mg/L), with a weight that was 2.03 times that of maternal *E. fuscoguttatus* at 15 months old [14]. EFT has the same karyotype as its parents (2*n* = 48, 2sm + 46t), and shows a closer growth pattern to paternal *E. tukula* through analysis of 20 morphological traits [15], which provides an excellent module to investigate the paternal-biased heterosis of groupers.

Fusion of two distant genomes in F1 hybrids leads to “genome shock” and “transcriptome shock,” which are described as extensive changes to patterns of parental gene expression [5]. In transcriptome analyses, overall expression of homologues is often used to reveal biological characteristics of hybrid groupers, such as Hulong grouper [16,17], Yunlong grouper [18,19], *E. coioides* ♀ × *E. lanceolatus* ♂ [20], and *E. fuscoguttatus* ♀ × *E*. *polyphekadion* ♂ [21]. With the availability of parental genomic resources, allele-specific expression (ASE) that hybrids preferentially express a particular allele from subgenomes under regulatory factors, has been suggested as another gene-regulatory mechanism of heterosis [22]. Biased subgenomic changes attributed to genotypic variation may help alleviate chaos from divergent genome mergers, and subsequent coordination may help exert the subgenome dominance in heterosis formation [2]. Increasing evidence in hybrid fishes has demonstrated that asymmetric allelic expression and subgenome dominance are pervasive and lead to phenotypic variations, including hybrid tilapia [5], *Megalobrama amblycephala* ♀ × *Xenocypri davidi* ♂ [1], and *M. amblycephala* ♀ × *Culter alburnus* ♂ [23,24]. However, knowledge regarding how subgenome interaction regulates genomic reconciliation and gene expression in hybrid groupers is limited.

Despite reciprocal F1 hybrids having the same genetic background, they exhibit substantial growth differences, which their epigenetic mechanism could explain [25]. Epigenetic modifications such as DNA methylation play crucial roles in gene expression, recombination, alternative splicing, and maintenance of subgenome dominance [26,27]. Recent studies have implicated that these parental differentially methylated regions (DMRs) most likely mediate the remodeling of methylation and transcriptional states at specific loci in the hybrids [28]. DNA methylation associated with growth heterosis has been studied in the hybrid snakehead fish (*Channa argus* × *C. maculata*) [29], the hybrids of *Carassius auratus* red var. and *Cyprinus carpio* L. [30], and the hybrid tilapia. For example, lower DNA methylation levels in the putative promoter region were negatively correlated with the elevated expression of *dgat2* in hybrid tilapia, which mediates the storage of fats for energy via catalyzing triglyceride synthesis [31]. However, whole-genome DNA methylation and its effects on the hybrid performance in EFT remains unknown.

Thus, the present study aimed to explore the paternal-biased heterosis in EFT through analysis of overall expression, ASE, subgenome dominance, and DNA methylation in three organs (pituitarium, liver, and muscle). Integrating transcriptome, whole-genome resequencing, and methylome data in EFT and maternal *E. fuscoguttatus*, providing valuable insights into the crucial pathways and genes involved in the heterosis of hybrid groupers.

## 2. Results

### 2.1. Identification of DEGs

**Figure 1:**
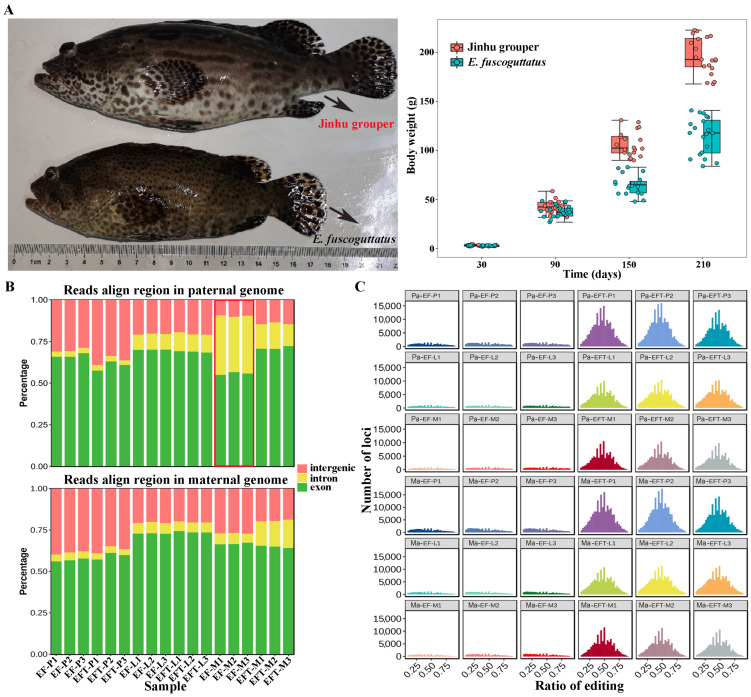
Comparing of growth traits and genomic alignments between groups. (**A**) Appearance and body weights of 7-month-old EFT and maternal *E. fuscoguttatus*. (**B**) The expressed reads mapping and (**C**) RNA editing analysis of groups against both parent genomes. EFT and EF represent Jinhu grouper and *E. fuscoguttatus*. P, L, and M represent pituitarium, liver, and muscle. Pa and Ma represent paternal and maternal genomic alignments, respectively. The red box represented the expressed reads were significantly mapped to introns against the reference genome.

The body weights of 7-month-old EFT and *E. fuscoguttatus* were 194.00 ± 39.03 g and 116.57 ± 34.53 g, with the ratio of 1.67 times (Figure 1A). RNA-seq was performed on pituitarium, liver, and muscle to explore the mechanism of growth heterosis involved in EFT compared with maternal *E. fuscoguttatus*. After filtering, a total of 897.90 million high-quality clean reads were obtained from 18 libraries. Meanwhile, their detailed statistics were presented in Table S2. When assembled transcripts were aligned to both parent genomes, the total mapped ratios of EFT ranged from 81.94% to 92.12%, and the other ranged from 71.59% to 95.93% for *E. fuscoguttatus*. Compared with pituitarium and liver, the highest mapping ratios (over 90.00%) were detected on the muscle across two tested fishes. The expressed reads of *E. fuscoguttatus* muscle were significantly mapped to introns against *E. tukula* reference genome (Figure 1B). RNA editing generates transcriptomic diversity, which was more common on hybrid transcriptome than maternal pure lines (Figure 1C).

**Figure 2:**
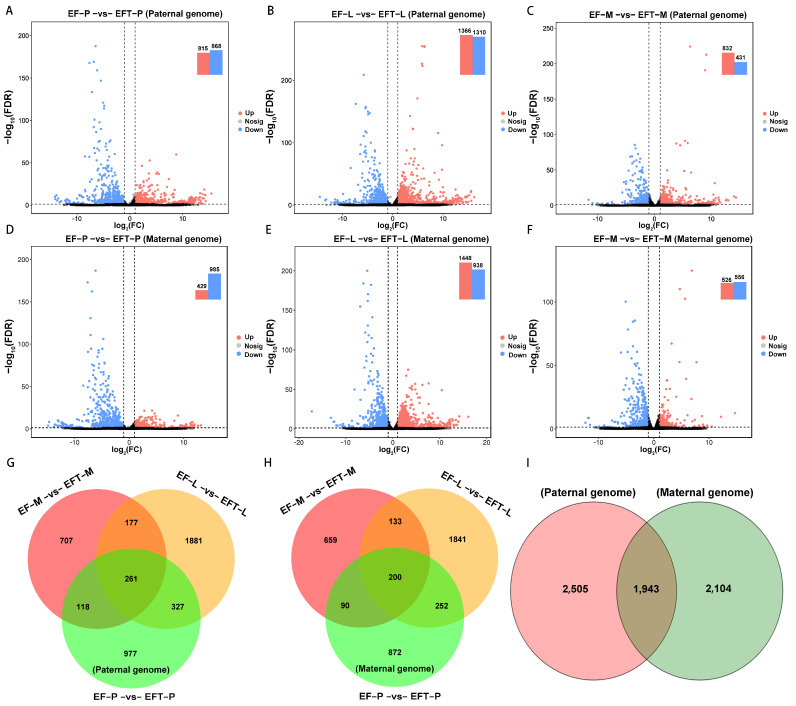
Comparing of differential expression volcano plots and Venn diagrams. (**A**–**F**) The identified DEGs among the three tested tissues between groups. (**G**–**I**) Venn diagram of the DEGs among different tissues and genome references. The dashed lines represented the threshold of significance. The black shaded areas indicated that these genes failed to achieve significant differences.

For paternal reference genome, 4448 DEGs were identified in EFT with the control of *E. fuscoguttatus*, which contains 261 shared genes across the three tissues, including 106 uniformly up-regulated and 150 all down-regulated genes. Residually, 5 shared genes did not show consistent up- or down-regulated expression trends (Figure 2A–C,G). Similarly, a total of 4047 DEGs were detected in the maternal genome, and 200 common genes were screened from the three tissues, including 13 all up-regulated genes, 182 all down-regulated genes, and 5 shared genes that failed to show consistent expression trends (Figure 2D–F,H). Based on the aforementioned homologous gene analysis, we found overlapped DEGS with 1943 and unique DEGs with 2505 and 2104, respectively, between paternal and maternal alignments (Figure 2I). Overall, EFT possessed more DEGs and up-regulated genes in the paternal genome alignment.

### 2.2. DEGs Were Affiliated to Many Amino Acid and Lipid Metabolism-Related Pathways

The KEGG enrichment analysis for DEGs demonstrated that amino acid metabolism, carbohydrate metabolism, lipid metabolism, and the digestive system were significantly enriched in the liver (*q* < 0.05), including these crucial pathways such as “Biosynthesis of unsaturated fatty acids”, “Fat digestion and absorption”, “Biosynthesis of amino acids”, and “Glycolysis/gluconeogenesis” (Figure S1A). Similarly, “ECM-receptor interaction” and “Cell adhesion molecules” involved in signaling molecules and interaction were significantly enriched in the pituitarium (Figure S1A).

Furthermore, KEGG enrichment analyses were independently performed on paternal (2505)- and maternal (2104)-unique DEGs (Figure S1B). Then, folding, sorting and degradation, and signal transduction were specially enriched in the paternal genome. Similarly, amino acid metabolism, carbohydrate metabolism, and the digestive system were solely enriched in the maternal genome. However, lipid metabolism, and signaling molecules and interaction were jointly enriched in the parental-unique DEGs, which contain “Biosynthesis of unsaturated fatty acids” and “Cell adhesion molecules” pathways.

### 2.3. Metabolic Pathways Was Significantly Enriched by Trend Analysis

A total of 4251 and 3834 DEGs screened from parental genomes were separately submitted for trend analysis. These tested DEGs were first enriched in “Metabolic pathways”, with a number of over 400 genes (Figure S2A,C). Subsequently, “Metabolic pathways” was also significantly enriched in the up-/down-regulated profiles of liver and muscle, as well as the consistently down-regulated profile with the order of pituitarium, liver, and muscle (Figure S2B,D). However, the consistently up-regulated profile showed significant enrichment in “PI3K-AKT signaling pathway”, “ECM-receptor interaction”, and “Focal adhesion” (Figure S2B,D). Furthermore, “Tight junction” and “Glycolysis/gluconeogenesis” were also specifically enriched in the muscle up-regulated profile (Figure S2B,D).

### 2.4. Candidate Modules Correlated to Growth Heterosis Were Identified by WGCNA

**Figure 3:**
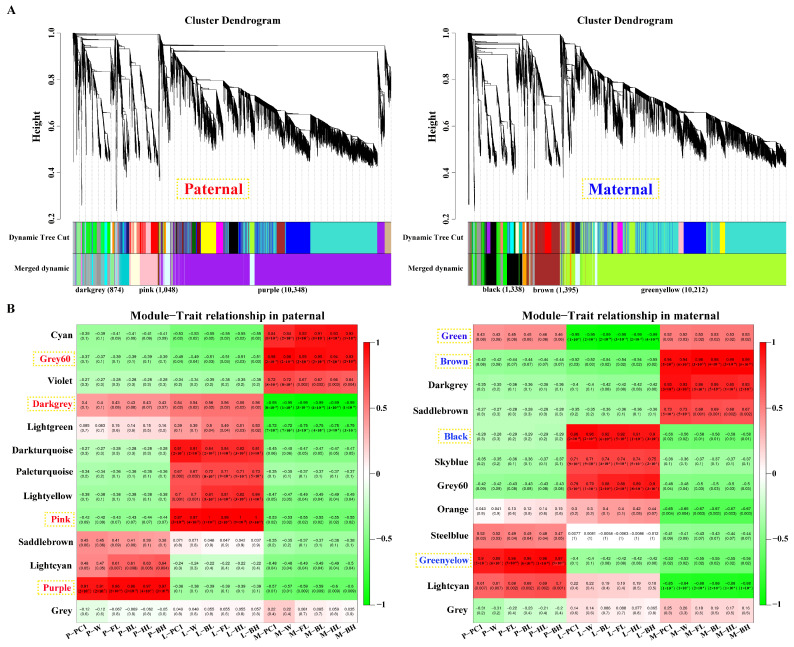
Screening of key modules by WGCNA. (**A**) Gene cluster dendrogram constructed from gene correlation coefficients. (**B**) Module–phenotype correlation analysis that the names of red and blue represent the most correlated modules in pituitarium, liver, and muscle against parental references. P, L and M represented pituitarium, liver and muscle. W, FL, BL, HL, and BH represented body weight, full length, body length, head length, and body height.

After filtering, 15,178 and 15,176 genes obtained from parental alignments were selected for a WGCNA, respectively. The gene cluster dendrogram was constructed based on the correlation coefficients of each gene expression (Figure 3A). Subsequently, 13 and 12 candidate modules were separately obtained with module sizes of 53 to 10,348. The calculation of module correlation coefficient and sample growth traits (Table S3) identified the most positive modules (*R*^2^ > 0.89) in the pituitarium, liver, and muscle including purple, pink and grey60 for paternal alignment, and green–yellow, black, and brown for maternal mapping. Furthermore, the most negative modules (*R*^2^ > 0.95) of dark–grey and green were detected in muscle and liver against paternal and maternal genome alignments, respectively (Figure 3B).

**Figure 4:**
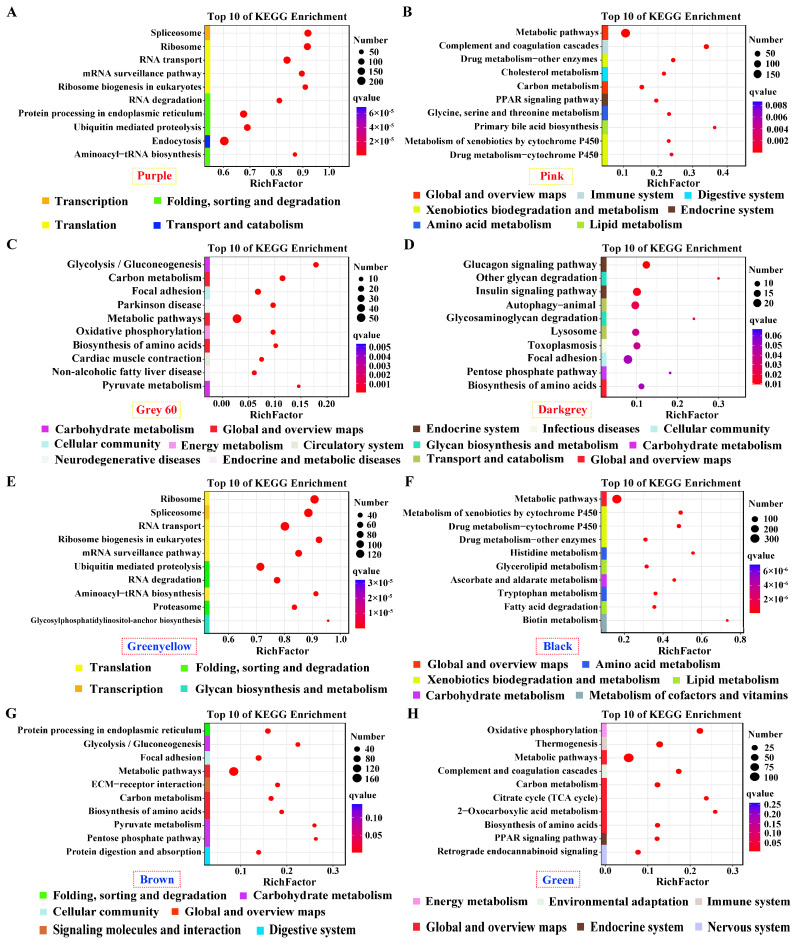
KEGG enrichment analysis for the significant module of growth traits. (**A**–**H**) The most correlated modules in pituitarium, liver, and muscle against paternal and maternal genome alignments. The red color text and the yellow dashed box represented paternal genome alignment, while the blue color text and the red dashed box represented maternal genome alignment. A larger circle represented more candidate genes were enriched.

Genes in positive modules of purple and green–yellow for pituitarium, possessing the most module sizes of over 10,000 genes, were significantly enriched in transcription; translation; and folding, sorting and degradation, which contain crucial pathways of “Spliceosome”, “Ribosom”, “RNA degradation”, and “Ubiquitin mediated pathway” (Figure 4A,E). Furthermore, “Metabolic pathways” was abundantly enriched in the positive/negative modules of liver, and the muscle-positive module (Figure 4B,C,F–H). Genes in “Biosynthesis of amino acids” displayed significant enrichment in muscle positive/negative modules, and liver negative module (Figure 4C,D,G,H). For muscle, “Glycolysis/gluconeogenesis” was solely enriched in the positive module. Additionally, “Glucagon signaling pathway” was identified in the negative module (Figure 4C,D,G). Finally, pathways related to lipid metabolism were enriched in liver-significant modules, including “Glycerolipid metabolism”, “Fatty acid degradation”, “TCA cycle”, and the “PPAR signaling pathway” (Figure 4B,F,H).

### 2.5. Identification of DMGs and KEGG Enrichment Analysis

A total of 3.22 × 10^9^ clean reads with the sequencing depth of 23.93× were obtained. Against paternal and maternal genome alignments, 76.61% and 74.67% evenly mapping rates were separately identified for EFT, while this was 61.78% and 84.40% for *E. fuscoguttatus* (Table S4). The genomic DNA methylation levels in the hybrid were 77.34%, 76.90%, and 75.21% for the pituitarium, liver, and muscle, respectively, which were all lower than pure *E. fuscoguttatus* (80.36%, 79.26%, and 79.83%). Moreover, 2 kb upstream regions exhibited the lowest DNA methylation levels across both groups (less than 60%), in which the level was higher in the hybrid compared to the pure line (Figure S3).

**Figure 5:**
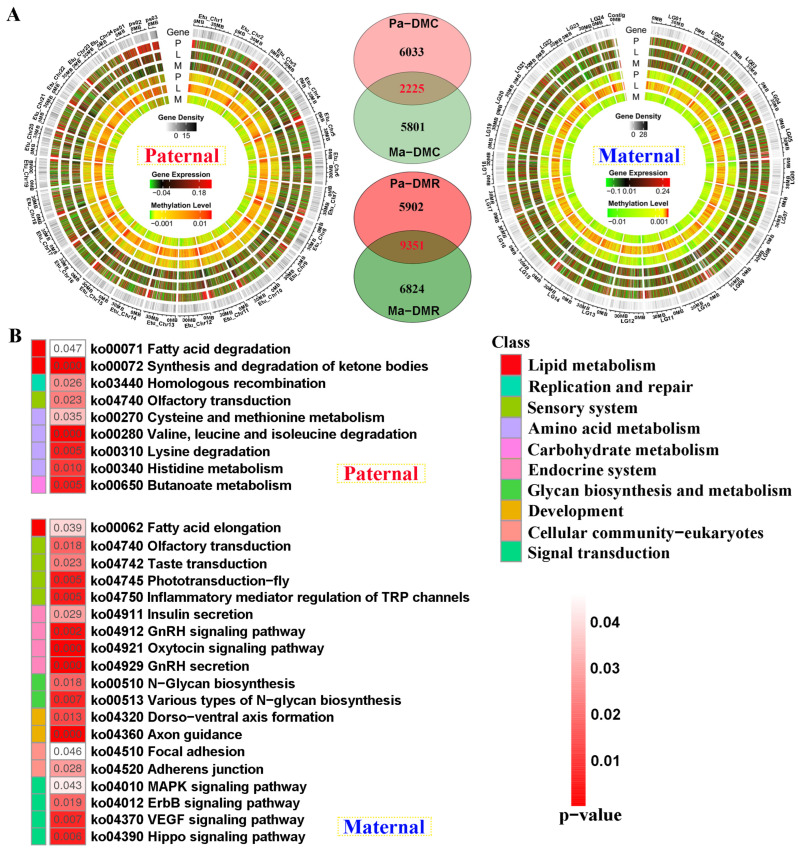
The genomic DNA methylation patterns and KEGG enrichment analysis of differentially methylated genes (DMGs) between groups. (**A**) The differentially methylated distribution by chromosome levels and Venn diagram of DMGs between paternal and maternal references. (**B**) KEGG results of the unique DMGs identified from paternal and maternal mappings. DMC and DMR severally represent differentially methylated cytosines and differentially methylated regions. P, L and M represented pituitarium, liver and muscle. Pa and Ma severally represented paternal and maternal genomic alignments. The red color text and the yellow dashed box represented paternal genome alignment, while the blue color text and the yellow dashed box represented maternal genome alignment.

Compared with *E. fuscoguttatus*, the distribution of differentially methylated and expressed regions was displayed by chromosomes in EFT against both genome alignments (Figure 5A). The total number of DMRs was higher in the male parent than that of the maternal mapping. Based on the screening criteria, 8258 and 8026 differentially methylated cytosines (DMCs)-related genes were separately identified for both references, including 2225 shared genes. Similarly, 15,253 and 16,175 DMR-related genes were revealed against parental references, including 5902 and 6824 unique DMGs, respectively (Figure 5A).

Contrary to transcriptome KEGG results, amino acid metabolism was solely enriched in paternal-unique DMGs (Figure 5B). However, endocrine system, development, and signal transduction were reflected in maternal DMGs, including “Insulin secretion”, and “GnRH secretion” pathways. Similarly, lipid metabolism was jointly enriched in these two references, which contained “Fatty acid elongation” and “Fatty acid degradation” pathways.

### 2.6. Correlation between Gene Expression and DNA Methylation

**Figure 6:**
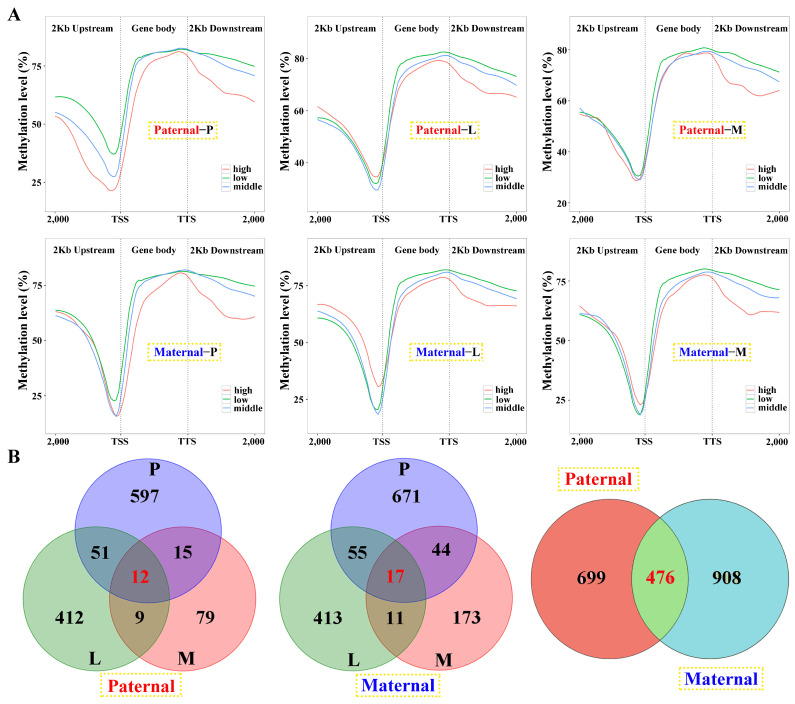
Linkage analysis of gene expression and DNA methylation within the samples against parental alignments. (**A**) The relationship between gene expression and DNA methylation of different regions in the samples. DEGs with low (1 < fpkm ≤ 10), middle (10 < fpkm ≤ 100) and high (fpkm > 100) expression were classified and their mean DNA methylation levels were calculated in the 2 kb upstream, gene body, and 2 kb downstream regions. (**B**) The Venn diagram of DMGs in three tested tissues and parental mappings. P, L and M represented pituitarium, liver and muscle. The red color text and the yellow dashed box represented paternal genome alignment, while the blue color text and the yellow dashed box represented maternal genome alignment.

Among the samples, negative correlations of gene expression and DNA methylation were revealed in the gene body and 2 kb downstream regions, and the latter was more obvious (Figure 6A). However, this correlation failed to be detected in the 2 kb upstream region, except for the pituitarium of paternal alignment. Common genes between DEGs and DMGs were extracted, which contained 1175 and 1384 for paternal and maternal references, within 476 overlapped genes (Figure 6B). The types of “E− and M+” and “E+ and M+” were mainly detected in paternal alignment, while “E+ and M−” and “E− and M−” were significant types of maternal mapping (Figure S4).

**Figure 7:**
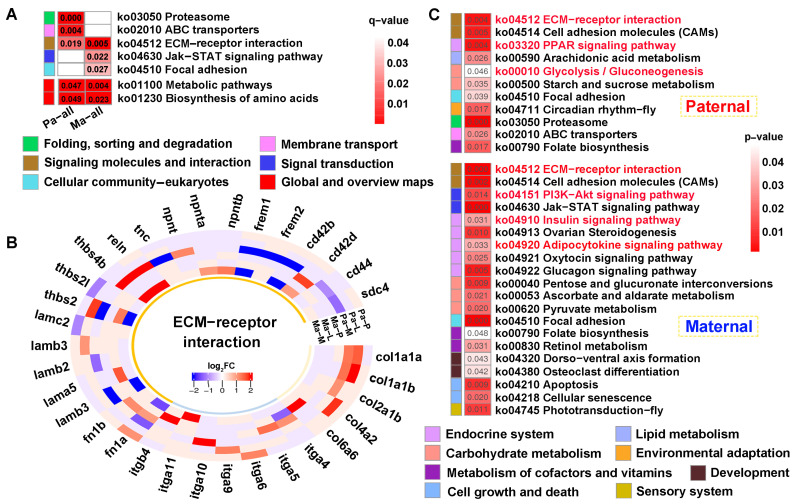
KEGG enrichment analysis of the common genes between DEGs and DMGs. (**A**) The functional classification of common genes within *q* < 0.05 against parental references, where “Metabolic pathway” and “Biosynthesis of amino acids” were enriched in *p* < 0.05. (**B**) The heatmap of ECM-receptor interaction-related genes within the tissues. (**C**) KEGG results of the specifically common genes from paternal and maternal references. The key pathways were shown in red font in (**C**). P, L and M represented pituitarium, liver and muscle. Pa and Ma severally represented paternal and maternal genomic alignments. The red color text and the yellow dashed box represented paternal genome alignment, while the blue color text and the yellow dashed box represented maternal genome alignment.

KEGG analysis of the common genes showed that “ECM-receptor interaction” was jointly enriched across parental references (*q* < 0.05) (Figure 7A). Thereinto, multiple gene families were up-regulated in this pathway, and *collagen* (*col1a1a*, *col1a1b*, *col2a1b*, *col4a2*, *col6a6*) and *integrin* (*itga4*, *itga5*, *itga6*, *itga9*, *itga10*, *itga11*, *itgb4*) genes were solely differentially expressed in paternal or maternal genome alignments (Figure 7B). Furthermore, “Metabolic pathways” and “Biosynthesis of amino acids” were also enriched in both references (*p* < 0.05) (Figure 7A). Finally, 699 and 908 unique genes screened from each reference were performed on KEGG analysis (*p* < 0.05) (Figure 7C). The results showed that signaling molecules and interaction was commonly identified in both mapping. However, signal transduction involving “PI3K-AKT signaling pathway” was specifically enriched in maternal alignment, inversely, and “Glycolysis/gluconeogenesis” was solely enriched in paternal mapping. Notably, pathways regulating glucose and lipid metabolism were broadly enriched, including the “Insulin signaling pathway”, “PPAR signaling pathway”, and “Adipocytokine signaling pathway”.

### 2.7. Paternal Subgenome Dominance Was Identified by ASE Analysis

**Figure 8:**
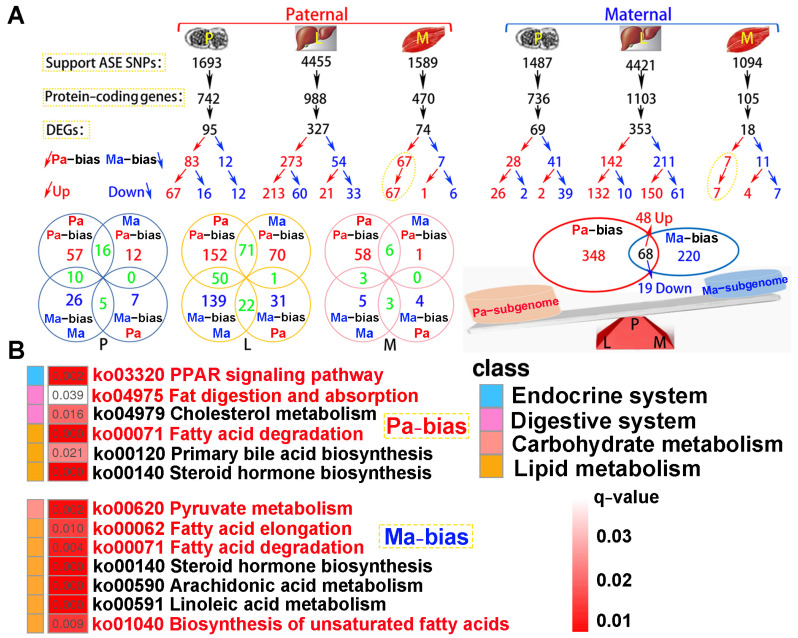
The identification of subgenome dominance and KEGG enrichment analysis. (**A**) Subgenome expression dominance in pituitarium, liver and muscle tissues, and the shared and specially homoeologous genes among different tissues and genome references. In the red triangle, the letters of P, L, and M represented these homoeologous genes coming from the three test tissues. (**B**) KEGG results of specifically subgenome bias DEGs from paternal and maternal references. The key pathways are shown in red font. ASE represented allele-specific expression. DEGs represented differentially expressed genes. P, L and M represented pituitarium, liver and muscle. Pa and Ma severally represented paternal and maternal genomic alignments. The red color text and the yellow dashed box represented paternal genome alignment, while the blue color text and the yellow dashed box represented maternal genome alignment.

A total of 4.06 × 10^8^ clean reads were obtained from parental whole-genome resequencing data (Table S5). Supporting ASE SNPs were identified across all tested tissues, subsequently corresponding protein-coding genes and DEGs were observed according to parental genome annotation (Figure 8A). For paternal alignment, 95, 327, and 74 subgenome bias DEGs were separately detected in the pituitarium, liver, and muscle. Notably, these bias DEGs presented paternal subgenome dominance not only on the total number but also on higher expression levels. However, for maternal mapping, only the total number supported maternal subgenome dominance, because numerous maternal bias DEGs in the liver were down-regulated compared to that of the hybrid offspring (150 vs. 61). Surprisingly, paternal bias DEGs screened from the muscle were wholly up-regulated in both reference genomes, and these bias DEGs from maternal alignment were almost overlapped on paternal mapping results. Through integrating the results of both references, a total of 416 paternal bias DEGs were detected, and this was greater than that of maternal bias 288 DEGs. Thereinto, 68 shared bias DEGs were co-located, including 48 up-regulated and 19 down-regulated homoeologous genes, and one gene of *tm4sf1* was up-regulated in the muscle while it was down-regulated in the liver.

KEGG analysis showed that subgenome bias genes were mainly enriched in lipid metabolism (*q* < 0.05) (Figure 8B). Thus, the “PPAR signaling pathway”, “Fat digestion and absorption”, and “Fatty acid degradation” were significantly enriched in paternal bias DEGs. Similarly, maternal bias DEGs were reflected on “Pyruvate metabolism”, “Fatty acid elongation”, “Fatty acid degradation”, and “Biosynthesis of unsaturated fatty acids”. Hence, “Fatty acid degradation” was jointly enriched in both subgenome results.

### 2.8. Lipid Metabolism Was Associated with EFT Heterosis

**Figure 9:**
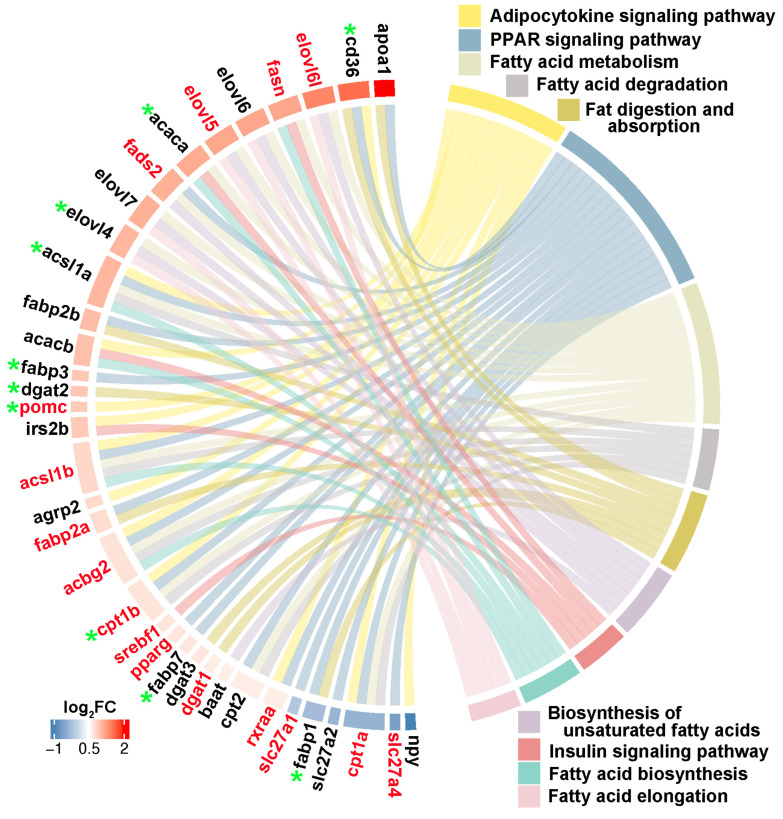
Lipid metabolism-associated genes identified in three somatotropic tissues. The red gene name represented DNA methylation, and the green star represented ASE regulation. The red to blue color bars at the outside circle represented the log_2_FC changes of expression values of these genes between groups. The different colors box represented lipid metabolism associated pathways, respectively.

**Figure 10:**
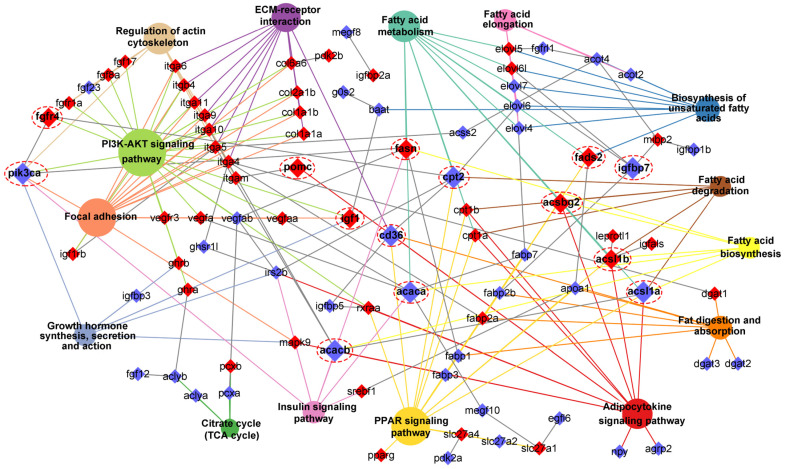
Interaction network of lipid metabolism-associated pathways and key genes. The different colors box and circle background represented associated genes and pathways, respectively. The red box genes represented DNA methylation, and the hub genes were magnified and noted by dashed red circles. If the gene belonged to the pathway, the connection line color was the same as the background color of the pathway.

Through integrating with transcriptome, methylome, and ASE results, we noticed that a number of genes involved in heterosis were mainly enriched in lipid metabolism-associated functional categories, like “Fatty acid biosynthesis”, “Fatty acid metabolism”, “Fatty acid elongation”, “Biosynthesis of unsaturated fatty acids”, “Fat digestion and absorption”, “Fatty acid degradation”, “PPAR signaling pathway”, “Adipocytokine signaling pathway”, and “Insulin signaling pathway”. The relationships of candidate genes engaged in the above functional categories were presented via various color ribbons, and found that numerous candidate genes were up-regulated, differentially methylated, and ASE regulated (Figure 9). To explore the regulatory network of these genes in lipid metabolism, interaction analyses were performed according to their expression patterns, and revealed that *fasn*, *fads2*, *igf1*, *igfbp7*, *pomc*, *cd36*, and *pik3ca* were recognized as hub genes (Figure 10).

**Figure 11:**
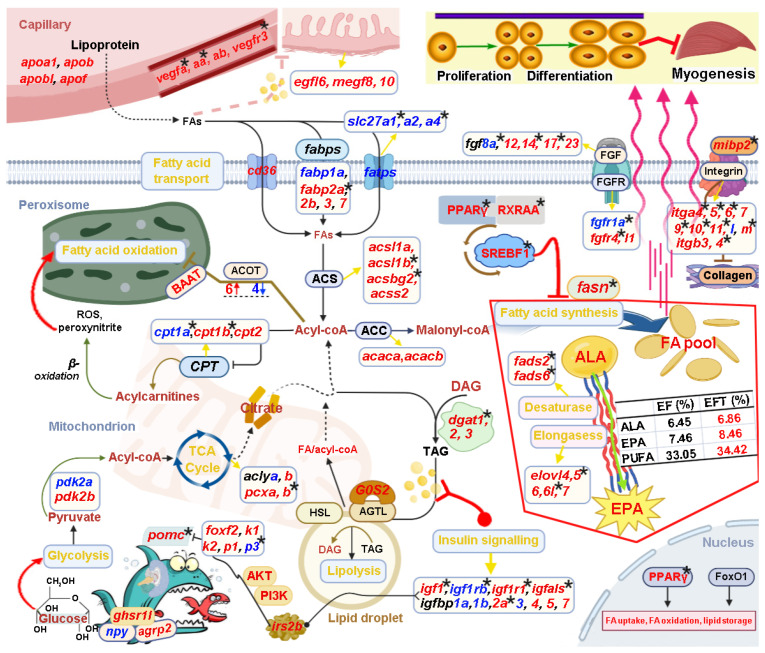
Schematic diagram reveals key genes and pathways involved in the heterosis formation of EFT. This regulatory network contained “Intake regulation”, “TCA cycle”, “Fatty acid synthesis”, “Fatty acid transport”, “Fatty acid oxidation”, “Lipolysis”, “EPA biosynthesis”, “Angiogenesis”, “Epidermal growth”, and “Myogenesis”. The up-regulated and down-regulated genes are shown in red and blue font, respectively. The star marks represented DNA-methylated regulation.

Based on the above bioinformatic analysis results in combination with manual literature searches about the functions of candidate genes, we constructed a potential transcriptional regulatory network of lipid metabolism for control of the heterosis formation in EFT (Figure 11). This regulatory network contained “Intake regulation” (*pomca*, *npy*, and *agrp2*), “TCA cycle” (*aclya*, *aclyb*, *pcxa*, and *pcxb*), “Fatty acid synthesis” (*fasn*, *pparg*, *rxraa*, and *srebf1*), “Fatty acid transport” (*cd36*, *slc27a*, *slc27a2*, and *slc27a4*), “Fatty acid oxidation” (*baat*, *cpt1a*, *cpt1b*, and *cpt2*), “Lipolysis” (*dgat1*, *dgat2*, *dgat3*, *lipe*, and *rbp4*), “EPA biosynthesis” (*fads2*, *fads6*, *elovl4*, *elovl5*, *elovl6*, *elovl6l*, and *elovl7*), “Angiogenesis” (*vegfa*, *vegfaa*, *vegfab*, and *vegfr3*), “Epidermal growth” (*egf16*, *megf8* and *vmegf10*), and “Myogenesis” (*fgf*, *fgfr*, *integrin*, and *collagen* gene family).

Notably, these identified genes were wholly up-regulated in “Fatty acid synthesis”, “EPA biosynthesis”, “Angiogenesis”, and “Epidermal growth”. Interestingly, the measurement of eicosapentaenoic acid (EPA) content in EFT was greater than maternal *E. fuscoguttatus* (8.46% vs. 7.46%) [32], which is consistent with the above results. Furthermore, a number of candidate genes were differentially methylated, such as *pomca*, *igf1*, *igfals*, *fasn*, *fads2*, *fads6*, *pparg*, *dgat1*, *fgf8a*, *fgf14*, *fgf17*, *fgfr1a*, and *fgfr4*; thereinto, “Fatty acid synthesis” and “Desaturase” are totally regulated by DNA methylation. In addition, 25 DEGs displayed subgenome bias in this regulatory network; thereinto, *fabp3*, *fabp7*, and *acot4* were co-located in both subgenome results, while they were totally up-regulated. Meanwhile, DMGs of *pomca*, *igfbp2a*, *fads6*, *cpt1b*, and *pcxb* also showed subgenome-biased expression. The detailed functional mechanisms of the genes are described in the Section 3.

### 2.9. Validation of RNA-Seq Data by a qRT-PCR Assay

**Figure 12:**
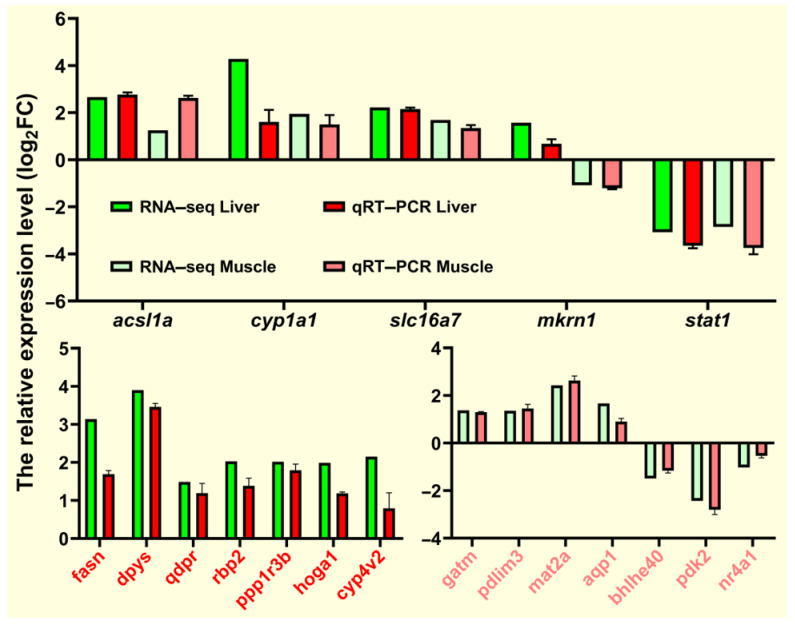
qRT-PCR for validation of transcriptome data. Expression levels of target genes were normalized to *β-actin* as a reference gene.

To verify the RNA-seq data, we selected 19 DEGs (*acsl1a*, *cyp1a1*, *slc16a7*, *mkrn1*, *stat1*, *fasn*, *dpys*, *qdpr*, *rbp2*, *ppp1r3b*, *hoga1*, *cyp4v2*, *gatm*, *pdlim3*, *mat2a*, *aqp1*, *bhlhe40*, *pdk2*, and *nr4a1*) from the liver and muscle tissues for qRT-PCR verification. The relative expression levels of these randomly selected genes are shown in Figure 12. The qRT-PCR expression trend of *fasn* and *acsl1a* involved in lipid metabolism was consistent with the RNA-seq expression trend and was similar to most genes. Overall, qRT-PCR results confirmed the reliability and accuracy of RNA-seq results.

## 3. Discussion

Heterosis is widely utilized in fish breeding and substantially contributes to high economic benefits on grouper cultivation. However, the utilization of heterosis far exceeds the level of theoretical research on this phenomenon. Although some relevant progress, such as classical genetics, molecular genetics, and epigenetics, has been obtained to explain its genetic basis, the mechanism by which heterosis forms remains obscure in hybrid groupers. Herein, transcriptome, whole-genome resequencing, and methylome integration was used to assess the paternal-biased heterosis in hybrid EFT.

### 3.1. Transcriptome and Methylome Patterns in the Hybrid and Purebred Groups

Compared with the pituitarium and liver, the muscle transcripts of maternal purebred were mainly mapped to introns in paternal reference genome. Meanwhile, the higher RNA editing ratio was detected on hybrid transcriptome than pure lines, and the catalyzed genes of adenosine deaminases (*adar*, *adarb1a*, and *adarb1b*) were all down-regulated in EFT. Furthermore, WGCNA analysis revealed that the genes in the positive-correlation modules of the pituitarium were significantly enriched in “Spliceosome”, “Ribosom”, “RNA transport”, “RNA degradation”, and “Aminoacyl-tRNA biosynthesis” pathways. The structural changes occurring in protein coding and non-coding regions may have profound impacts on heterosis formation through multiple mechanisms, including modifying splice sites, affecting RNA nuclear export, and altering microRNA sequences or their target sites [33].

Moreover, the genomic DNA methylation levels detected on three somatotropic tissues were all lower in the hybrid grouper than the purebred parent value, indicating that significant extensive demethylation had occurred. The correlation between DNA hypomethylation and heterosis had been reported in various hybrid species [29,30]. In *Larix kaempferi* reciprocal hybrids, the heterotic hybrids showed a lower genomic DNA methylation level; however, the non-heterotic hybrids displayed a close DNA methylation level to the midparent value [34]. The de novo and maintenance of DNA methylation in mammals require DNA methyltransferase (DNMTs) and DNA methylation hydroxylase (TETs). Herein, the transcript levels of *dnmt3b* and *tet1* were all up-regulated in the liver of EFT (Figure S5). The up-regulated *dnmt3b* was DNA hypomethylation, showing a negative correlation regulation. However, *tet1* did not exhibit statistically significant differential DNA methylation.

### 3.2. The Relationship between Transcriptome and Methylome of EFT

The results indicated that the up-regulated gene expressions were negatively correlated to their DNA hypomethylation in the gene body and 2 kb downstream, while this correlation was indistinctive in 2 kb upstream, which might be attributed to its lower methylation levels. It has been reported that 2 kb upstream (the promoter region) as a repressive epigenetic mark had a role in down-regulating gene expression. The DNA hypomethylation at the promoter region might promote the higher transcript expression of genomic DNA [35]. Similarly, the sharply-decreased methylation in the gene body can regulate transcript elongation or affect splicing [25]. Furthermore, KEGG results displayed that amino acid metabolism was solely enriched in paternal-unique DMGs, whereas it was solely enriched in maternal-unique DEGs.

### 3.3. Paternal Subgenome Dominance Might Cause Paternal-Biased Heterosis in EFT

The merger of divergent genomes routinely leads to one subgenome to become dominant over the other subgenome, resulting in subgenome bias in gene content and expression. Uncovering the underlying mechanisms in subgenome dominance provides great opportunities for agricultural, ecological, and evolutionary research [36]. The body size of grouper varies greatly, and the heterotic hybrids frequently exhibited paternal-biased growth heterosis, providing an ideal template for probing the molecular basis of subgenome regulation in hybrid lineages. This study found that ASE analysis supported paternal subgenome dominance not only on gene numbers but also on higher transcript levels. An unexpected and exciting finding was that paternal bias DEGs were wholly up-regulated in the muscle of EFT. Furthermore, transcriptome also revealed the total numbers of DEGs, up-regulated genes, and paternal-unique DEGs, were all significant in paternal genomic alignment. Undoubtedly, paternal subgenome dominance was largely a cause of heterosis formation in EFT, while detailed mechanisms involved in growth regulation need further exploration. A growing body of evidence supports the role that homoeologous exchange might play in subgenome dominance [36,37]. Herein, a total of 68 DEGs were co-located in both parental subgenome bias results, while many of them were up-regulated, indicating that homoeologous exchange and recombination existed in EFT hybrid genome.

### 3.4. Lipid Metabolism Associated with EFT Heterosis Formation

Multi-omics-integrated results highlighted the role of lipid metabolism in EFT heterosis formation. Fatty acids function both as energy source and as signals for metabolic regulation, acting through enzymatic and transcriptional networks to modulate gene expression, growth, and survival pathways, and inflammatory and metabolic responses [38]. We constructed a potential transcriptional regulatory network of lipid metabolism for control of the heterosis formation in EFT.

### 3.5. “Fatty Acid Synthesis” Was Significantly Enriched in EFT

Pathway-related genes (*fasn*, *pparg*, *rxraa* and *srebf1*) were wholly up-regulated, and showed differential DNA methylation. The master regulator in adipogenesis is the peroxisome proliferator-activated receptor gamma (PPARγ), and by heterodimerization to retinoid X receptor (RXRα), the PPARγ-RXRα dimer regulates transcription of downstream target genes [39]. PPARγ was first identified as a transcription factor integral to adipocyte differentiation, playing a crucial role in the triacylglycerol synthesis, angiogenesis, and skeletal muscle [40,41]. Most normal mammal tissues preferentially use dietary (exogenous) lipid for synthesis of new structural lipids, whereas de novo (endogenous) fatty-acid synthesis, as another important source of energy and anabolism, is commonly detected in the proliferating cancer cells and the undifferentiated stem cells [42,43]. The elevated glycolysis produced an excess of the pyruvate, which was further converted to acetyl-CoA for de novo fatty-acid synthesis (Figure 11). Thereinto, acetyl-CoA carboxylase (ACC) and fatty-acid synthase (FASN) were considered as two key enzymes, and they were up-regulated in EFT. In short, ACC carboxylates acetyl-CoA to form malonyl-CoA, subsequently, FASN converted the malonyl CoA product to long-chain fatty acids [42,44]. Therefore, dominantly adopting de novo synthesis for cellular fatty-acid accumulation, like cancer cells and stem cells, might explain the reason for the heterosis formation in EFT, through affecting fundamental cellular processes, including signal transduction and gene expression.

### 3.6. Up-Regulated Synthetic Pathways Might Explain the Higher EPA Content in EFT

For “EPA biosynthesis”, desaturases and elongases are the enzymes responsible for the conversion of α-linolenic acid (ALA, C18:3*n*−3) to eicosapentaenoic acid (EPA, C20:5*n*−3). Desaturases are responsible for the double bond formation between two carbons leading to more unsaturated fatty acids. Subsequently, elongases catalyzes the elongation of the aliphatic chain of carbons to produce long-chain polyunsaturated fatty acids (LC-PUFAs) [45]. EPA as a vital member of LC-PUFAs is implicated as a protective agent in a range of pathologies such as cardiovascular disease and metabolic syndrome, and appears to be particularly important for cognitive and behavioral function [46]. Startlingly, the EPA content in EFT muscle was greater than maternal *E. fuscoguttatus* (8.46% vs. 7.46%) [32], and these key enzymes encoded by the *FADS* and *ELOVL* gene family were mostly up-regulated and differentially methylated, such as *fads2*, *fads6*, *elovl5*, and *elovl6l* genes. Thereinto, *fads6* presented DNA hypomethylation and paternal bias up-regulated expression.

### 3.7. Triglyceride Synthesis by Dgats Was Dominant in EFT Adipocytes

Triglycerides provide the major storage form of fatty acids for metabolic fuel and membrane building blocks, which is catalyzed by diacylglycerol acyltransferase (DGAT) [47]. DGAT catalyzes the glycerol phosphate pathway, and the monoacylglycerol pathway is considered the main pathway of triglyceride synthesis in cells. Therefore, DGAT facilitates the joining of a diacylglycerol (DAG) with a fatty acyl CoA, resulting in the formation of triglyceride [48]. Herein, the *dgat*s gene family was wholly up-regulated, and *dgat1* was down-regulated by DNA methylation. Inversely, when energy supply is limited, lipolysis of adipocyte triglycerides is used for producing hydrolyzed fatty acids for metabolic fuel to other tissues [47]. Interestingly, these promoting adipocyte lipolysis genes were all down-regulated in EFT, including the rate-limiting enzyme hormone-sensitive lipase (*lipe*) [49], and adipocyte-secreted retinol-binding protein 4 (*rbp4*) that exhibit maternal bias down-regulated expression [50]. Therefore, these molecular regulators might uncover the phenomenon that the crude fat content in EFT was greater than maternal *E. fuscoguttatus* (4.2% vs. 3.0%) [32].

### 3.8. Lipids as Signaling Molecules Regulate Multiple Biological Functions in Heterosis Formation

Signaling lipids such as fatty acids, DAG, and sphingolipids regulate insulin sensitivity directly by modulating the insulin receptor (IR), insulin receptor substrate (IRS), PI3K, and AKT, and indirectly by altering the flux of substrates through lipogenesis, lipolysis, and lipid oxidation [51]. Chain length and the degree of desaturation of the fatty acid moieties increase the complexity of biological roles in lipid molecules. PUFAs, in particular, the essential EPA and docosahexaenoic acid (DHA), are anti-inflammatory and have been associated with improved insulin sensitivity in animal studies [51,52,53]. Furthermore, increasing insulin stimulates PI3K signaling in POMC and AgRP neurons, resulting in a K_ATP_ channel-dependent hyperpolarization and electrical silencing [54]. We showed that *igf1*, *igfals*, *igf1rb*, *igflr1*, and *irs2b* were mostly up-regulated, and differentially methylated. Moreover, “PI3K-AKT signaling pathway” was significantly enriched in the consistently up-regulated profile by the trend analysis. The *igf1* gene, a key factor of the GH-IGF system, can promote cell growth and proliferation, and regulate fuel metabolism peripherally, which was co-identified as a crucial regulator in Hulong grouper and Yunlong grouper transcriptome analyses [16,19]. Herein, the up-regulated *igf1* co-existed with hypomethylation and hypermethylation regions in the gene body, but its DNA methylation level was overall down-regulated.

### 3.9. Heterosis Analysis in EFT Gonad Development, Low-Temperature Tolerance, and Hypoxia Tolerance

Our research showed that the gonad of EFT developed normally but was weaker than maternal *E. fuscoguttatus,* as well as for the development rates and the volume of oocytes. Furthermore, the serum estradiol (E2), 11-keto testosterone (11-KT), testosterone (T), and progesterone (P) levels of hybrids were obviously lower than those of purebreds at 10, 18, 24, and 36 months [55]. So far, some of sperms were obtained from a few mature F1 hybrid EFT, but no females have been found to lay eggs. The present results found that numerous genes involved in gonad development were down-regulated and differentially methylated, such as *hsd17b3*, *spats2*, *fstl3*, and *fstl5* (Figure S3). Thereinto, the spermatogenesis-associated genes of *hsd17b3* and *spats2* were generally down-regulated across the tested tissues, and presented DNA hypermethylation. For low-temperature tolerance, the stopped feeding and the half-lethal temperature of EFT decreased by 2–3 °C compared with those of maternal *E. fuscoguttatus* [14]. Comparative transcriptome analysis suggested that lipid metabolism, especially the PPAR signaling pathway, played a positive role in the response to temperature stress in EFT, and a great number of *hsps* (*hsp30*, *hsp70*, *hsp90aa1*, *hsp90b1*, *hspa4*, *hspa5*, *hspa8*, *hspa9*, and *hspa13*) were differentially expressed [56]. We found *hsp70*, *hspa4*, and *hspa13* exhibited lower expression levels and DNA hypermethylation under normal farming conditions. Similarly, the hypoxia tolerance gene of *hif1a* also displayed down-regulated expression and hypermethylation. These findings provide valuable clues for further dissecting the molecular bases of heterosis underlying stress tolerance and gonadal diapause in EFT.

## 4. Materials and Methods

### 4.1. Resources and RNA Extraction

In April 2020, EFT and *E. fuscoguttatus* half sibs families were correspondingly constructed via artificial fertilization. All F1 larval fish were raised under indoor industrial conditions at Laizhou Ming Bo Aquatic Co., Ltd. (Laizhou, China), with a water temperature of 24 ± 2 °C, dissolved oxygen of 6.48 ± 0.30 mg/L, pH of 7.33 ± 0.06, and salinity of 28.52 ± 0.30‰. A compound pelleted diet (Santong Bioengineering Co., Ltd., Weifang, China) was fed at 8 a.m. and 4 p.m. every day. Then, 20–30 randomly selected progenies were measured for body weight, full length, body length, head length, and body height at each sampling point. After 7 months of cultivation, three somatotropic tissues from the pituitarium, liver, and muscle were isolated from nine individuals in triplicate and immediately stored in liquid nitrogen.

Each triplicate sample was pooled together as one mixed sample, generating 18 samples hereafter named EF-P1-3, EF-L1-3, and EF-M1-3 for *E. fuscoguttatus*, EFT-P1-3, EFT-L1-3, and EFT-M1-3 for EFT. Total RNA was extracted using the Trizol Reagent Kit (Invitrogen, Carlsbad, CA, USA) according to the manufacturer’s protocol.

### 4.2. Library Construction and Sequencing

RNA quality and concentration were assessed using an Agilent 2100 Bioanalyzer (Agilent Technologies, Santa Clara, CA, USA) and by electrophoresis on a 1% agarose gel, then the total RNAs with RIN > 7.0 were used for library construction. Briefly, the enriched mRNA was fragmented into short fragments using a fragmentation buffer and reverse-transcribed into cDNA by using the NEBNext Ultra RNA Library Prep Kit for Illumina (New England Biolabs, Ipswich, MA, USA). The purified cDNA fragments were end repaired, poly(A) added, and ligated to Illumina sequencing adapters. After size selection, the resulting cDNA libraries were sequenced on Illumina Novaseq6000 by Gene Denovo Biotechnology Co. (Guangzhou, China).

### 4.3. Read Processing and Analysis of Differentially Expressed Genes (DEGs)

Clean reads were obtained by filtering the adapters and low-quality sequences using fastp v0.18.0 software, while rRNA was removed using Bowtie2 v2.2.8. The remaining cleaned reads were separately mapped to *E. tukula* (PRJNA745015) and *E. fuscoguttatus* (AP022675–AP022699) reference genomes using Hisat2 v2.1.0 with default parameters. For each transcription region, a FPKM (fragment per kilobase of transcript per million mapped reads) value was calculated to quantify expression abundance, using the RSEM software package v1.3.3.

The DEGs were identified using DESeq2 software 1.44.0, with the threshold of adjusted *p*-value < 0.05 and |log_2_FoldChange| ≥ 1. To infer the potential functions of the identified DEGs, GO and KEGG enrichment analyses were performed with the threshold of *p*-value < 0.05.

### 4.4. Trend Analysis

To examine the expression patterns of DEGs in EFT, expression data for each sample (in the order of pituitarium, liver, and muscle) were normalized and then clustered by Short Time-series Expression Miner (STEM) [57]. The DEGs in specific profile were subjected to GO and KEGG enrichment analyses.

### 4.5. Weighted Gene Co-Expression Network Analysis (WGCNA)

Co-expression networks were constructed using WGCNA to find biologically significant modules associated with paternal-biased heterosis in EFT [58]. Briefly, expression values across multiple samples were normalized and subjected to R package of WGCNA to identify co-expression modules, with the power 16 and minModuleSize 50. Subsequently, module eigengenes were analyzed to determine the correlations between modules and the growth traits (body weight, full length, body length, head length, body height, and their first principal component (PC1)). Modules with the highest/lowest Pearson correlation coefficients and *p* < 0.05 were considered positively/negatively related, while the genes manifesting the closest relationships with other genes tended to be hub genes. KEGG enrichment analyses were conducted to for genes in candidate modules to understand the biological functions. The network for key genes characterization was visualized using Cytoscape_3.9.1 [59].

### 4.6. Genomic DNA Library Construction and Bisulfite Sequencing

For interpreting the underlying epigenetic mechanism involved in the heterosis of fast growth, the same tissues for RNA-seq were resubmitted for whole-genome DNA methylation analysis. The integrity of the extracted DNA was checked via a NanoPhotometer^®^ spectrophotometer (IMPLEN, München, Germany), and by agarose gel electrophoresis. Thereafter, DNA was fragmented to 100–300 bp by Sonication (Covaris, Inc., Woburn, MA, USA), and purified with MiniElute PCR Purification Kit (QIAGEN, Valencia, CA, USA). The DNA fragments were then processed by end repair, poly(A) added, and ligated to methylated sequencing adapters. Subsequently, the ligated DNA was converted with bisulfite using the Methylation-Gold Kit (ZYMO Research, Irvine, CA, USA); unmethylated cytosine was converted to uracil during sodium bisulfite treatment. Finally, the products were PCR amplified and sequenced on an Illumina HiSeq^TM^ 2500 (Gene Denovo Biotechnology Co., Guangzhou, China).

### 4.7. Data Filtering and Methylation Level Analysis

Raw bisulfite sequencing data were filtered by removing adaptor sequences, low-quality reads, and contamination. The clean reads were separately aligned against the paternal (PRJNA745015) and maternal (AP022675–AP022699) genomes using BSMAP v2.90 [60]. The methylated cytosines were called using a Perl script and tested with a previous correction algorithm [61]. The methylation level was calculated based on methylated cytosine percentage in the whole genome, on each chromosome, and at different genomic regions in CG, CHG, and CHH contexts.

### 4.8. The Identification of Differentially Methylated Genes (DMGs)

DMRs between two samples were determined using methylKit v1.7.10 with a calling window size of 200 bp and the minimum read coverage of 4 bp. DMRs for each sequence context (CG, CHG, and CHH) were based on the following criteria: (1) For CG and CHG, numbers in each window ≥ 5, absolute values of the difference in methylation ratio ≥ 0.25, and *q* ≤ 0.05; (2) for CHH, numbers in a window ≥ 15, absolute values of the difference in methylation ratio ≥ 0.15, and *q* ≤ 0.05; (3) for all C, numbers in a window ≥ 20, absolute values of the difference in methylation ratio ≥ 0.2, and *q* ≤ 0.05. Subsequently, KEGG pathway enrichment analyses were performed for DMR-related genes.

### 4.9. The Correlation of DNA Methylation and Gene Expression in Samples

Genes were categorized into four classes based on their expression levels to determine the correlation between gene transcription and DNA methylation. These classes included non-expressed (RPKM ≤ 1), low-expressed (1 < RPKM ≤ 10), middle-expressed (10 < RPKM ≤ 100), and high-expressed groups (RPKM > 100). Spearman’s correlation analysis was performed to discern the statistical relationships between DNA methylation and gene expression within ±2 kb flanking regions and the gene body.

Common genes were extracted between DMGs and DEGs, and their KEGG enrichment analysis was conducted to explore the potential functions of DNA methylation responsible for DEGs.

### 4.10. SNP Calling and ASE Analysis

Genomic DNA was extracted from each parental individual for DNA library construction, using the Paired-End DNA Sample Prep Kit (Illumina Inc., San Diego, CA, USA). Then, whole-genome resequencing was completed on an Illumina Novaseq6000 sequencing platform by Gene Denovo Biotechnology Co., (Guangzhou, China). These raw reads were firstly filtered to remove reads that had ambiguous bases greater than 10% of the total bases, then reads over 50% of the length were less than Q20, and reads aligned to the adapters. The clean reads were mapped to the paternal (PRJNA745015) and maternal (AP022675–AP022699) genomes using BWA with the settings “mem 4 −k 32 −M” [62]. The BAM file was used for SNP detection by the GATK “HaplotypeCaller” function. Variant call format (VCF) files were generated after quality filtering (QD < 2.0 || FS > 60.0 || MQ < 40.0, GQ < 20). For paternal and maternal resequencing samples, SNPs aligned to the paternal genome with the 0|0 × 1|1 type, and 1|1 × 0|0 SNPs mapped to the maternal genome were used for ASE analysis, respectively.

Two sets of parental genome sequences were first aligned using the reciprocal BLAST (BLASTP, v. 2.2.26) with an e-value cut off of 1 × 10^−5^ to identify orthologs. A total of 17,361 orthologs were obtained for subsequent analysis. Then, the mapped reads in hybrid transcriptions were divided into two categories based on the parental specific SNPs. Parental-specific reads/maternal-specific reads were used to detect homeolog expression bias [23]. If the ratio ≥ 2 and *q* ≤ 0.01 (in three biological replicates), the expression level of the gene was considered biased to the paternal subgenome, whereas it was considered biased to the maternal subgenome if the ratio ≤ 0.5 and *q* ≤ 0.01. Subsequently, KEGG pathway enrichment analyses were separately performed for paternal or maternal bias DEGs.

### 4.11. Quantitative Reverse Transcription Polymerase Chain Reaction (qRT-PCR)

To verify the reliability of sequencing data, 19 DEGs were randomly selected from the transcriptome data for EFT and *E. fuscoguttatus*. The total RNAs from liver and muscle tissues were reverse transcribed to synthesize cDNA by using the PrimeScript™ RT Reagent Kit with gDNA Eraser (Takara Bio, Kusatsu, Japan). qRT-PCR was performed on triplicate technical replicates and the *β-actin* gene [56] was used as the internal control for normalization of gene expression. The reaction system included 2 μL of template cDNA, 0.8 μL of forward primer, 0.8 μL of reverse primer, 10 μL of TB Green Premix Ex Taq II (Tli RNaseH Plus) (Takara Bio, Kusatsu, Japan), and 6.4 μL of RNase-Free ddH2O. qRT-PCR was performed on LightCycler480II (Roche, Basel, Switzerland) and the amplification conditions were as follows: pre-denaturation at 95 °C for 30 s, followed by 40 cycles of 95 °C for 5 s, and 60 °C for 30 s. Then, relative quantification was performed, and melting curve analysis was used to verify the generation of a single product at the end of the assay. The fold changes in the relative expression of these genes were analyzed using the 2^−ΔΔCt^ method, and the log_2_FC value was calculated. The sequences of the primers used are given in Table S1.

## 5. Conclusions

EFT provides an excellent model for exploring the genetic and molecular basis of paternal-biased growth heterosis from the genomic level. Here, multi-omics-integrated results highlighted the role of genomic DNA methylation, and paternal subgenome dominance in heterosis formation. Enrichment analysis showed that these candidate genes were mainly involved in “Fatty acid synthesis”, “EPA biosynthesis”, “Triglyceride synthesis”, “Signaling lipids”, and the “PI3K-AKT signaling pathway”. These results might contribute to explaining how hybrid grouper present paternal-biased heterosis, increasing our understanding of grouper cross breeding.

## Data Availability

The transcriptome, methylome, and whole-genome resequencing data presented in the study are deposited in the NCBI SRA repository (https://www.ncbi.nlm.nih.gov/ (accessed on 2 July 2024)), accession number PRJNA1020269, PRJNA1020917, and PRJNA1020990, respectively.

## Supplementary Materials

The following supporting information can be downloaded at: https://www.mdpi.com/article/10.3390/ijms25179740/s1.
