# Supplementary material for: DNA Methylation and Subgenome Dominance Reveal the Role of Lipid Metabolism in Jinhu Grouper Heterosis
Source: Int J Mol Sci. 2024 Sep 9;25(17):9740. doi: 10.3390/ijms25179740 (PMC11396105; doi:10.3390/ijms25179740)
Supplement: Supplementary file 1 [file ijms-25-09740-s001.zip › Supplementary Figures.pdf]

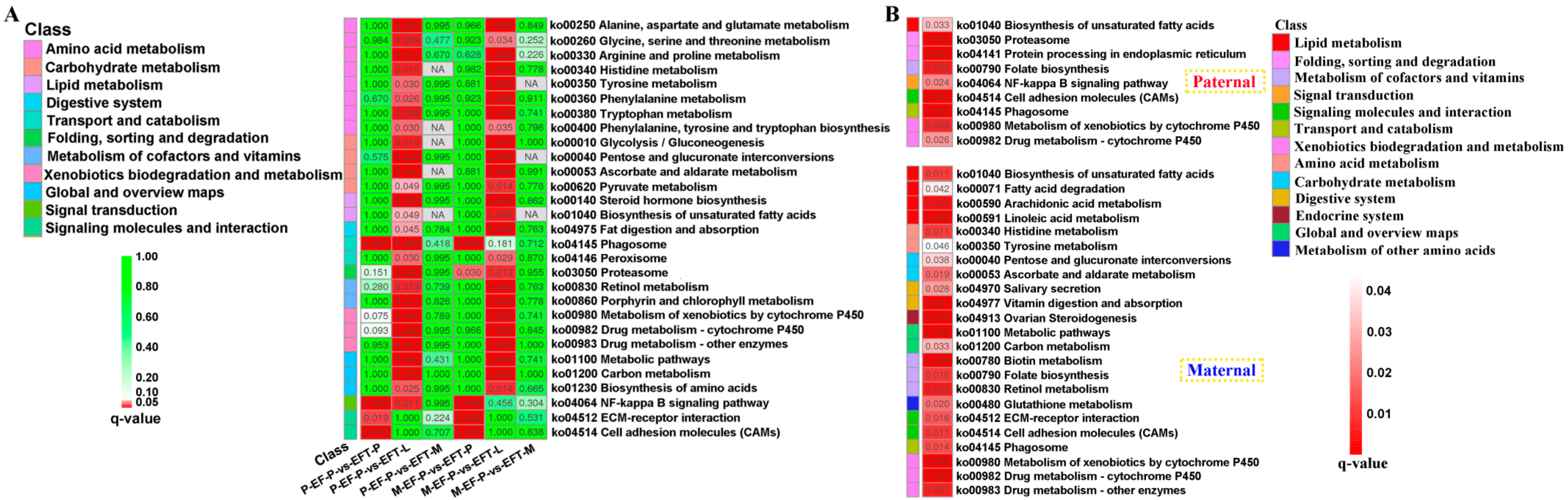

Figure S1. The KEGG enrichment for the identified DGEs.

(A) KEGG enrichment results of the comparison groups among various tissues and references. (B) KEGG results of the unique DEGs identified from paternal and maternal references, respectively.

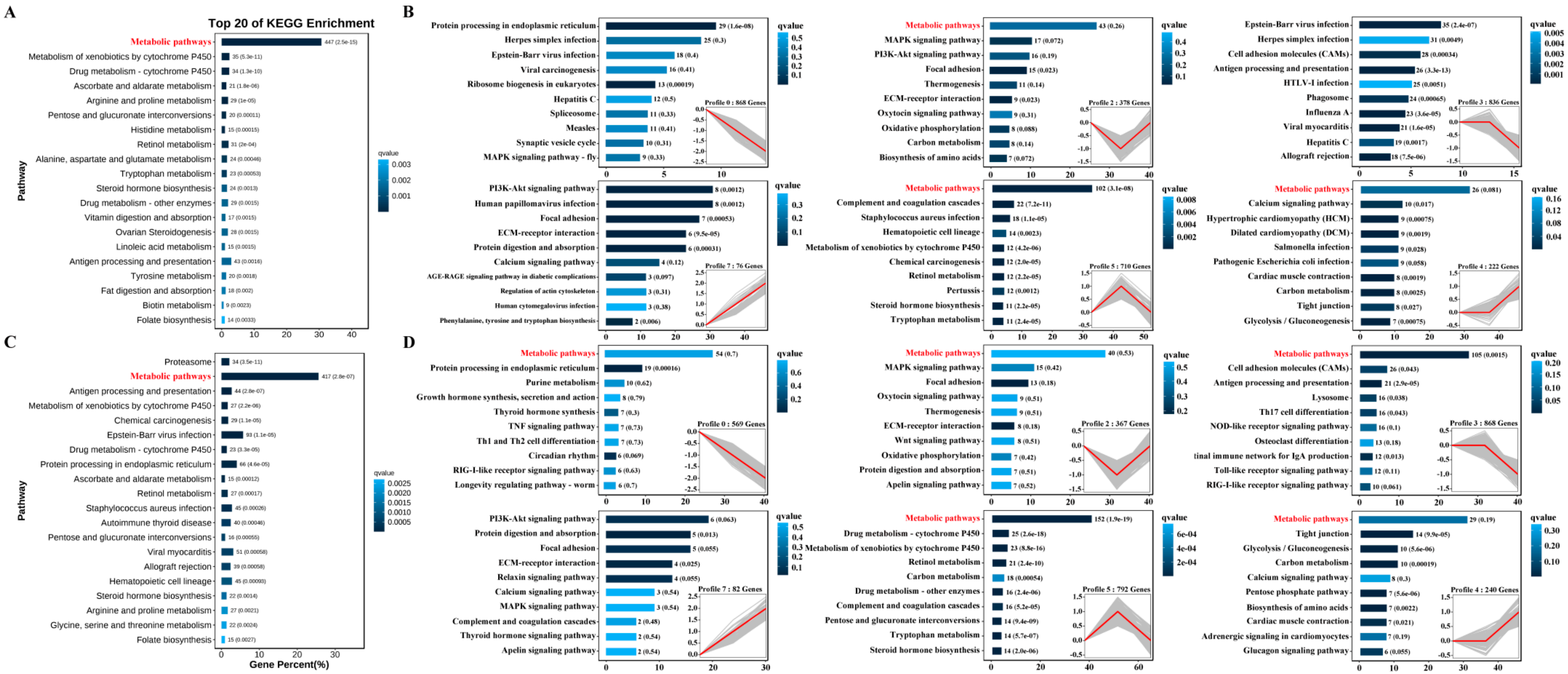

Figure S2. Trend analysis and KEGG enrichment of all transcripts based on pituitarium, liver, and muscle from Jinhu grouper. (A and C) The identified genes for trend analysis from paternal and maternal alignments. (B and D) The KEEG results of significant profiles against paternal and maternal references, respectively. “Metabolic pathways” was significantly enriched and shown in red font.

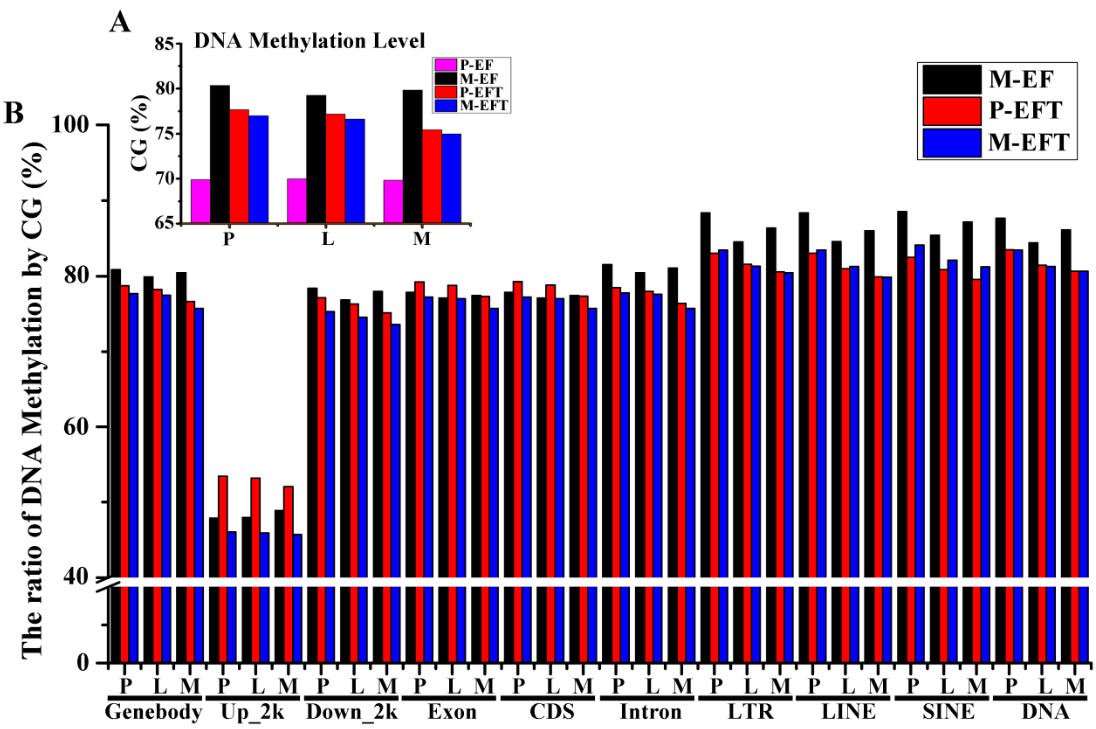

Figure S3. The DNA methylation patterns in three somatotropic tissues by different genomic regions.

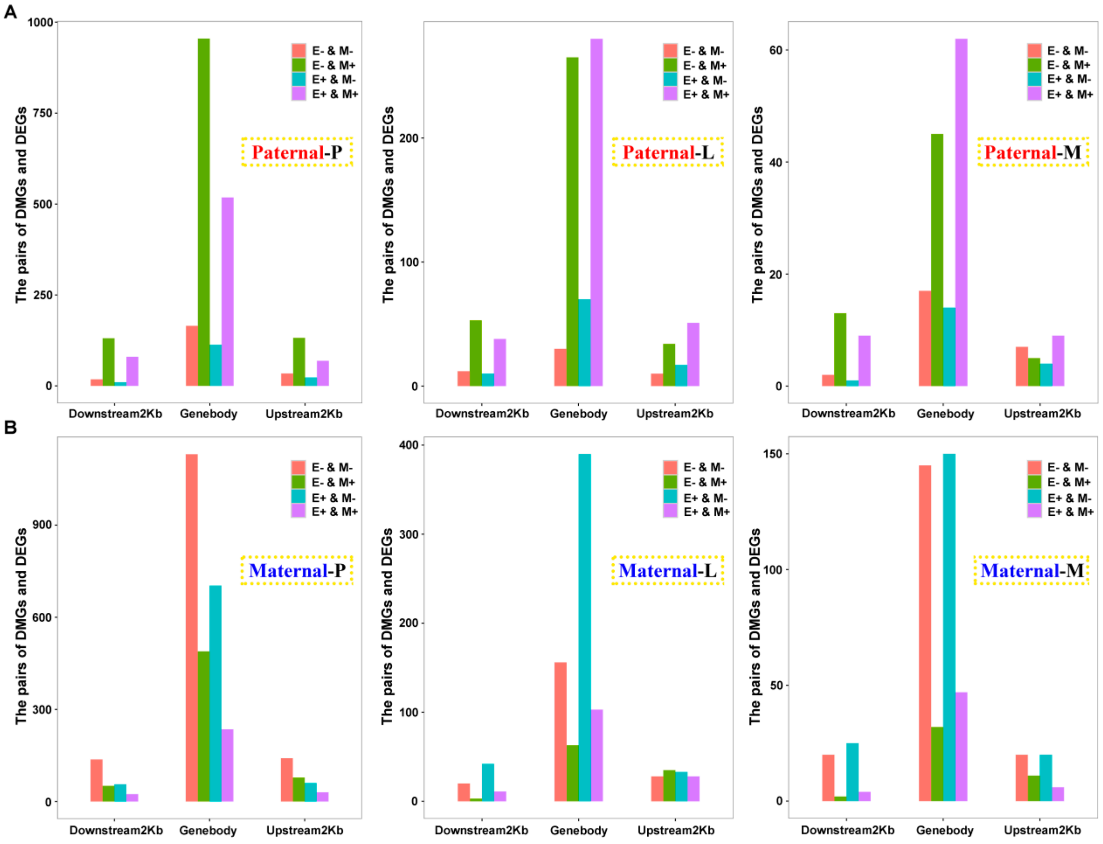

Figure S4. The trends calculation between DMGs and DEGs within the samples against paternal (A) and maternal (B) alignments.

Gene	Annotation	P-P	P-L	P-M	M-P	M-L	M-M
dnmt3b	DNA (cytosine-5)-methyltransferase 3b		1.37			2.14	
hif1a	Hypoxia-inducible factor 1-alpha	-1.00			-1.30		
hsp70	Hsp70 family protein	-1.66		2.93			
hspa4	Heat shock 70 kDa protein 4		-1.16				
hspa13	Heat shock 70 kDa protein 13		-1.65			-1.13	
hsd11b2	Hydroxysteroid 11-beta dehydrogenase 2					2.52	
hsd17b3	Testosterone 17-beta-dehydrogenase 3	-1.17	-1.96		-1.16	-1.57	
hsd17b8	Estradiol 17-beta-dehydrogenase 8						1.39
spats2	Spermatogenesis-associated serine-rich protein 2		-2.30	-1.62			
fstl3	Follistatin-related protein 3	-4.32					
fstl5	Follistatin-related protein 5				-1.40		
esr1	Estrogen receptor alpha				-2.11		
esr2	Estrogen receptor beta	-2.05	1.54		-2.11	1.87	
ovch2	Ovochymase 2	-1.89	4.25				

2 1 0 -1 -2

Figure S5. Expression of candidate genes involved in reproduction and stress tolerance.
